# Stratifying Breast Lesion Risk Using BI-RADS: A Correlative Study of Imaging and Histopathology

**DOI:** 10.3390/medicina61071245

**Published:** 2025-07-10

**Authors:** Sebastian Ciurescu, Simona Cerbu, Ciprian Nicușor Dima, Victor Buciu, Denis Mihai Șerban, Diana Gabriela Ilaș, Ioan Sas

**Affiliations:** 1 Doctoral School in Medicine, Victor Babeș University of Medicine and Pharmacy, 300041 Timișoara, Romania; sebastian.ciurescu@umft.ro (S.C.); victor.buciu@umft.ro (V.B.); 2 Department of Obstetrics and Gynecology, Victor Babeş University of Medicine and Pharmacy, 300041 Timișoara, Romania; denis.serban@umft.ro (D.M.Ș.); sas.ioan@umft.ro (I.S.); 3 Department XV of Orthopaedics, Traumatology, Urology and Medical Imaging, Discipline of Radiology and Medical Imaging, Victor Babeș University of Medicine and Pharmacy, 300041 Timisoara, Romania; 4 Department VI Cardiology, Division of Cardiovascular Surgery, Victor Babeș University of Medicine and Paharmacy, 300041 Timisoara, Romania; dima.ciprian@umft.ro; 5 Department of Medical Semiology, Victor Babeș University of Medicine and Pharmacy, 300041 Timișoara, Romania; diana_ilas@yahoo.com

**Keywords:** BI-RADS, breast cancer, mammography, ultrasound, histopathology, immunohistochemistry, lesion stratification, Ki-67, ER/PR, HER2

## Abstract

*Background and Objectives*: The accuracy of breast cancer diagnosis depends on the concordance between imaging features and pathological findings. While BI-RADS (Breast Imaging Reporting and Data System) provides standardized risk stratification, its correlation with histologic grade and immunohistochemical markers remains underexplored. This study assessed the diagnostic performance of BI-RADS 3, 4, and 5 classifications and their association with tumor grade and markers such as ER, PR, HER2, and Ki-67. *Materials and Methods*: In this prospective study, 67 women aged 33–82 years (mean 56.4) underwent both mammography and ultrasound. All lesions were biopsied using ultrasound-guided 14G core needles. Imaging characteristics (e.g., margins, echogenicity, calcifications), histopathological subtype, and immunohistochemical data were collected. Statistical methods included logistic regression, Chi-square tests, and Spearman’s correlation to assess associations between BI-RADS, histology, and immunohistochemical markers. *Results*: BI-RADS 5 lesions showed a 91% malignancy rate. Evaluated features included spiculated margins, pleomorphic microcalcifications, and hypoechoic masses with posterior shadowing, and were correlated with histological and immunohistochemical results. Invasive tumors typically appeared as irregular, hypoechoic masses with posterior shadowing, while mucinous carcinomas mimicked benign features. Higher BI-RADS scores correlated significantly with increased Ki-67 index (ρ = 0.76, *p* < 0.001). Logistic regression yielded an AUC of 0.877, with 93.8% sensitivity and 80.0% specificity. *Conclusions*: BI-RADS scoring effectively predicts malignancy and correlates with tumor proliferative markers. Integrating imaging, histopathology, and molecular profiling enhances diagnostic precision and supports risk-adapted clinical management in breast oncology.

## 1. Introduction

Breast cancer remains the most frequently diagnosed cancer among women worldwide and is a leading cause of cancer-related mortality. Accurate diagnosis of breast cancer is essential in reducing mortality, particularly in the context of global disparities in access to timely imaging and biopsy. Early detection through imaging and tissue diagnosis plays a critical role in improving prognosis, guiding therapeutic decisions, and optimizing patient outcomes. Among the available imaging modalities, mammography remains the cornerstone of routine breast cancer screening, often supplemented by breast ultrasound (echography) to enhance lesion characterization, especially in dense breast tissue. Given the known association between mammographic density and breast cancer-specific mortality, its integration into risk models has gained traction [1]. This is supported by evidence showing that beyond breast density, imaging-based risk measures play an increasing role in personalized screening pathways [2]. BI-RADS, developed by the American College of Radiology, provides a standardized lexicon and risk stratification tool for interpreting breast imaging findings. By categorizing lesions based on radiologic suspicion for malignancy, BI-RADS facilitates consistent clinical decision-making across radiologists and institutions. However, the prognostic power of BI-RADS lies in its alignment with not only histological confirmation but also biological tumor behavior. Also, the true diagnostic value of BI-RADS scoring relies on its concordance with histopathological findings obtained through biopsy or surgical excision. Correlating breast tumor imaging findings with the histopathological report is a crucial step in breast cancer diagnosis and disease management. This radiologic–pathologic verification is particularly relevant for intermediate BI-RADS categories, where uncertainty may otherwise delay diagnosis or overtreat benign disease. This process is essential not only for confirming malignancy but also for ensuring accurate lesion characterization in staging, treatment planning, and post-treatment surveillance. Imaging typically identifies abnormalities such as masses, calcifications, or architectural distortions. Correlation with histopathology verifies whether the imaging abnormality corresponds to a benign or malignant lesion. This step reduces the risk of false reassurance in the case of missed cancers and minimizes overtreatment of benign lesions. When imaging and pathology findings are discordant, further steps—such as additional biopsy, surgical excision, or multidisciplinary case review—are necessary to resolve diagnostic uncertainty.

Despite the widespread clinical use of BI-RADS, relatively few studies have systematically evaluated its predictive value in real-world settings by linking imaging scores with histological subtype, tumor grade, and proliferation indices. Understanding these relationships can enhance the diagnostic performance of imaging and inform more nuanced risk stratification. Moreover, identifying radiologic indicators that correlate with molecular markers such as Ki-67 or hormone receptor status may offer insights into tumor biology even before biopsy is performed.

The present study aims to investigate the relationship between BI-RADS scores and histopathological features in a cohort of patients referred for breast imaging and biopsy in a Romanian tertiary care center. Furthermore, by analyzing proliferation indices (Ki-67) and receptor status in relation to imaging scores, we aim to explore whether radiological suspicion may serve as a proxy for tumor aggressiveness. Specifically, we evaluate how radiological classification aligns with histological diagnosis, tumor grade, and immunohistochemical markers, and explore whether patient demographics, such as age, further refine this association. By doing so, we seek to contribute to the optimization of diagnostic workflows in breast cancer evaluation and highlight the importance of rigorous radiologic–pathologic correlation in daily clinical practice.

## 2. Materials and Methods

### 2.1. Study Design and Setting

This prospective, single-center, observational study was conducted over a 14-month period, from December 2023 to March 2025, in the Radiology and Imaging Department of the Municipal Emergency Clinical Hospital in Timișoara, Romania. All diagnostic procedures: mammography, ultrasound, biopsy, and histopathologic evaluation, were defined prior to patient enrollment. Patients were consecutively included during the study period based on pre-established inclusion criteria. While no long-term clinical follow-up was performed, all data were recorded prospectively during the diagnostic process. The study was initiated following the conclusion of a three-year national breast cancer screening program, during which a working unit established within the hospital evaluated women aged 50–69 years who were enrolled in the national initiative. At the end of the organized screening campaign, our imaging unit remained active, transitioning to an opportunistic screening model that accepted patients of all ages for breast evaluation.

Standard imaging protocols were applied according to age and clinical presentation. Asymptomatic patients under the age of 40 were evaluated by ultrasound, while those aged 40 and above underwent mammographic examination. All biopsied cases included in the present analysis had previously undergone both mammography and ultrasound, with BI-RADS scores of 3, 4, or 5 assigned based on combined imaging findings. BI-RADS scoring was performed by board-certified breast radiologists with subspecialty experience. During the study period, a total of 986 patients accessed the imaging service. Of these, 67 patients underwent breast biopsy due to the identification of suspicious lesions. Biopsies were performed under ultrasound guidance using a 14-gauge tru-cut needle. Imaging assessments included digital 2D and 3D mammography using the GE Pristina platform, and breast-targeted ultrasound performed with a high-resolution probe dedicated to breast pathology, also provided by General Electric. In accordance with institutional clinical protocols, patients with a family history of breast or ovarian cancer in first-degree relatives were referred for genetic counseling. Additionally, contrast-enhanced breast magnetic resonance imaging (MRI) was selectively employed in specific clinical scenarios following histopathological confirmation of breast cancer, including preoperative staging, evaluation of multifocality, and monitoring of the therapeutic response to neoadjuvant chemotherapy. MRI was selectively applied and not part of the standard diagnostic algorithm. It was performed in a limited number of cases for preoperative staging or neoadjuvant planning. These cases were excluded from statistical correlation analyses to avoid introducing bias, and MRI data were not used in BI-RADS scoring or lesion classification.

### 2.2. Ethical Approval

The study protocol was reviewed and approved by the Ethics Committee of the Victor Babeș University of Medicine and Pharmacy and the affiliated hospitals (approval number 43/20 October 2023). All procedures were conducted in accordance with the Declaration of Helsinki. Patient confidentiality was ensured through data anonymization and secure handling of sensitive information.

### 2.3. Study Population and Data Collection

Eligible participants were adult female patients referred for breast imaging between December 2023 and March 2025 who, at the time of evaluation, received a BI-RADS 3, 4, or 5 classification based on mammographic and ultrasonographic findings. Inclusion in the study was based on imaging suspicion of malignancy, prior to biopsy or final histopathological diagnosis. Patients were prospectively enrolled at the time of imaging, and subsequently underwent ultrasound-guided 14-gauge core biopsy. Only those with complete histopathological and imaging data, and who provided informed consent, were retained in the final analysis. Exclusion criteria included the absence of BI-RADS documentation, incomplete histopathological evaluation, or refusal to provide informed consent.

**Figure 1:**
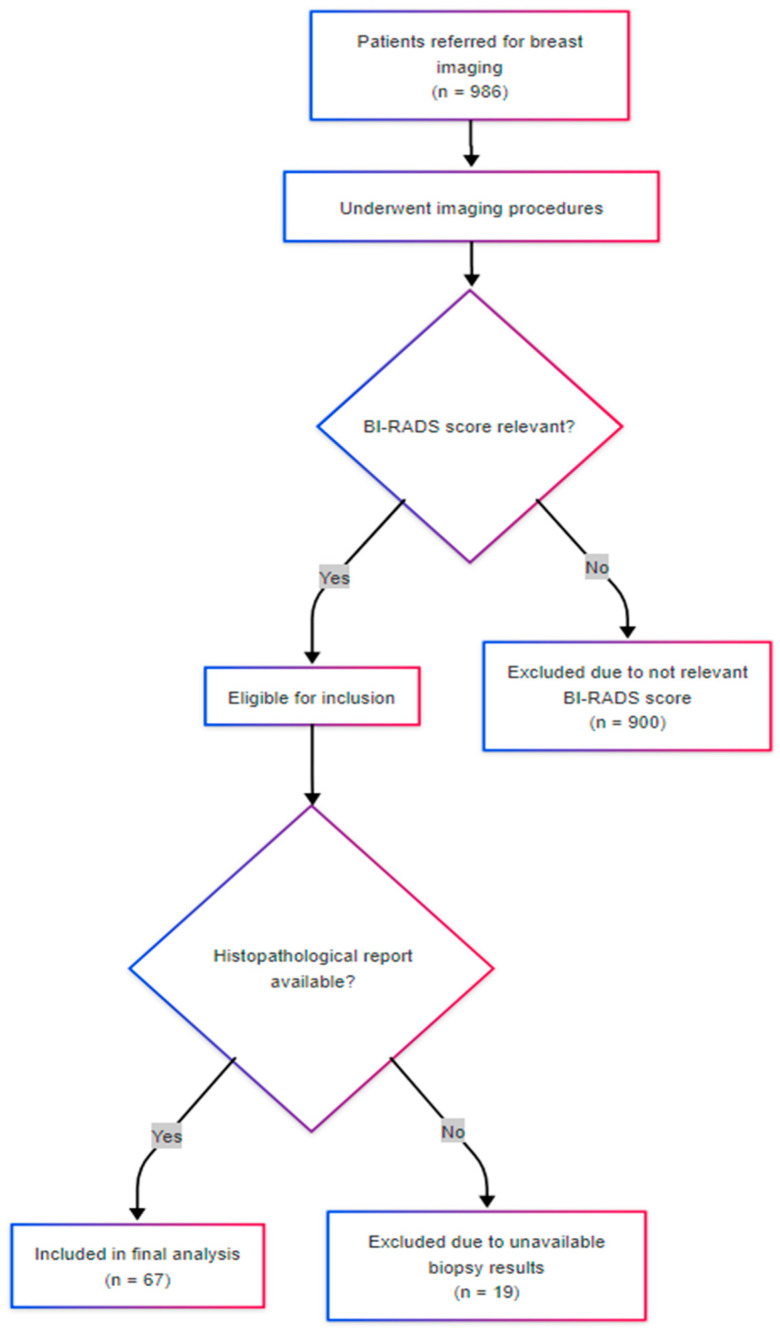
Flowchart illustrating the patient selection process. From 986 patients referred for breast imaging between 2023 and 2025, only those with relevant BI-RADS scores and available histopathological reports were included in the final analysis (n = 67). Patients were excluded due to non-relevant BI-RADS categories or unavailability of biopsy results.

For each patient, data were recorded regarding age, residence, BI-RADS classification, histological diagnosis and grade, and, where available, immunohistochemical markers including estrogen receptor (ER), progesterone receptor (PR), HER2 status, and Ki-67 proliferation index. Histologic diagnoses were classified into five diagnostic groups: malignant neoplasms, benign proliferative lesions, benign non-proliferative lesions, atypical lesions with malignant potential, and inconclusive findings. This grouping was based on histological descriptors and aligned with established pathological criteria to enhance statistical interpretability while preserving clinical relevance. Figure 1 illustrates the patient inclusion process. Of the 986 individuals screened during the study period, 67 met the inclusion criteria and were analyzed.

### 2.4. Statistical Analysis

Data collection and curation was performed using Microsoft Excel 2021 and advanced statistical analyses were conducted using JASP (v0.19.3). Descriptive statistics were calculated for all continuous and categorical variables, including mean, standard deviation, and frequency distributions.

Categorical associations were assessed using Chi-square tests or Fisher’s exact test, as appropriate. These were used to explore the relationship between BI-RADS scores, histologic diagnosis groups, and other categorical variables such as place of residence and histologic grade. For continuous variables such as age and Ki-67 index, differences between diagnostic groups (benign vs. malignant) were evaluated using Mann–Whitney U tests.

Spearman’s rank correlation coefficients were calculated to assess monotonic relationships between continuous variables, including BI-RADS scores, Ki-67 index, and hormone receptor expression levels. Logistic regression modeling was used to evaluate the predictive power of the BI-RADS score for malignancy classification, with performance metrics including accuracy, sensitivity, specificity, and area under the receiver operating characteristic (ROC) curve. Due to the modest sample size and potential for model overfitting, only the BI-RADS score was included as a predictor in the logistic regression. Future studies with larger datasets could incorporate age, lesion morphology, and molecular subtype to refine model accuracy. The final logistic model’s parameters were interpreted using Wald tests. A significance threshold of *p* < 0.05 was used for all inferential statistics.

## 3. Results

A total of 67 patients meeting the inclusion criteria were analyzed to explore associations between radiological (BI-RADS) scores, histopathological outcomes, and demographic and immunohistochemical variables. The mean age of the study cohort was 56.46 years (SD = 14.13), with a range from 33 to 82 years. While breast density, clinical symptoms, and family history were variably documented in imaging records, this information was not systematically collected and is therefore not included in the current analysis. These clinical variables will be addressed in future iterations of the diagnostic protocol. Among the 14 cases for which Ki-67 data were available, the mean proliferation index was 29.93% (SD = 19.58), indicating considerable variability in tumor aggressiveness. The limited number of Ki-67 observations and the high standard deviation limit the generalizability of this result, which should be interpreted with caution. Correlations involving Ki-67 are therefore considered exploratory in nature.

**Figure 2:**
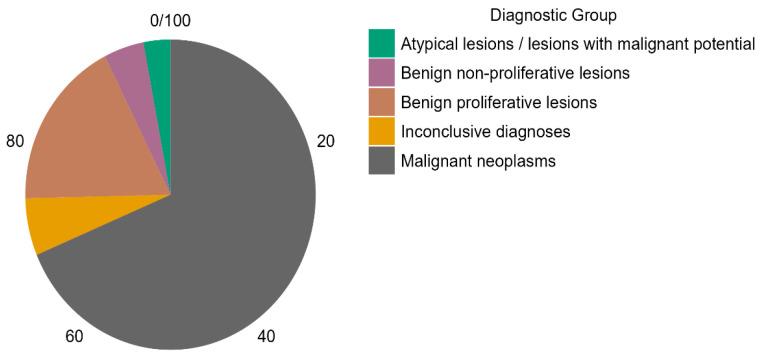
Distribution of diagnostic groups among patients undergoing mammographic and histopathological evaluation (n = 67). Malignant neoplasms comprised the largest category, followed by benign proliferative lesions.

**Figure 3:**
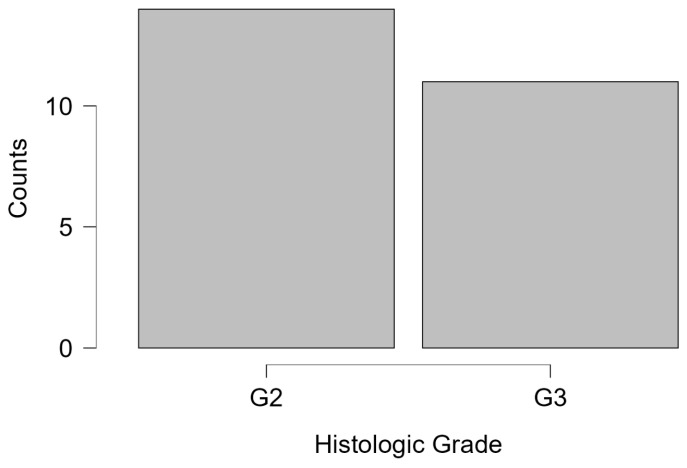
Histogram illustrating the distribution of histologic grades (G2 and G3) among evaluable cases (n = 25). A majority were graded G2, suggestive of moderate differentiation.

Histological categorization revealed that the majority of patients (68.7%) were diagnosed with malignant neoplasms, followed by benign proliferative lesions (17.9%), benign non-proliferative lesions (4.5%), atypical lesions with malignant potential (3.0%), and inconclusive diagnoses (6.0%). Missing data were handled through case-wise exclusion from specific sub-analyses. For example, histologic grading was available for only 25 of 32 malignant cases, and Ki-67 data were present in 14 cases. These subsets were analyzed independently and were not imputed. The six percent of cases with inconclusive diagnoses were retained for overall frequency reporting but excluded from comparative or inferential analyses due to diagnostic uncertainty. These distributions are visually represented in Figure 2. The histologic grade was available in 25 cases, with 56% graded as G2 and 44% as G3. Notably, G1 cases were absent, reflecting the predominance of intermediate-to-high-grade malignancies in the studied population (Figure 3). The absence of G1 histologic grades may reflect either a referral bias toward more suspicious lesions in this diagnostic setting or the limited sample size. This finding could also suggest underrepresentation of indolent tumors in opportunistic screening contexts, warranting further investigation in population-based studies.

**Figure 4:**
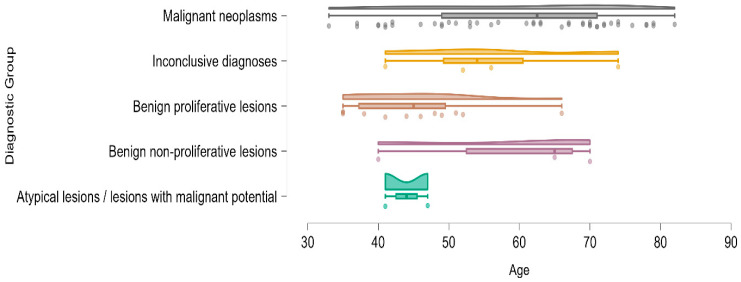
Raincloud plot illustrating the age distribution across diagnostic categories. Each layer represents the individual patient values, density estimation, and central tendency for the five diagnostic groups. Patients with malignant neoplasms exhibited higher mean ages compared to those with benign or atypical findings, supporting the observed statistical significance.

Age also differed significantly across diagnostic groups, with ANOVA revealing a significant between-group effect (F = 3.549, *p* = 0.011). Patients diagnosed with malignant lesions tended to be older on average compared to those in benign or atypical categories. This age-related distribution is further visualized in the raincloud plot shown in Figure 4, which demonstrates a clear upward shift in mean age among malignant neoplasms relative to benign and atypical groups.

Contingency table analysis revealed a significant association between BI-RADS scores and diagnostic category (χ^2^ likelihood ratio = 37.35, df = 8, *p* < 0.001), confirming that increasing BI-RADS scores corresponded to a higher probability of malignancy. Specifically, 30 of the 33 patients with a BI-RADS 5 score were confirmed to have malignant histology, supporting the clinical validity of this radiologic stratification (Table 1).

To further validate this relationship, Kendall’s Tau-b was computed and yielded a strong positive correlation (τ = 0.722, Z = 5.212, *p* < 0.001), suggesting that higher BI-RADS scores are consistently aligned with more severe histologic outcomes.

A binary logistic regression was conducted to assess the predictive utility of BI-RADS scores in distinguishing benign from malignant lesions. The model including BI-RADS as a predictor (Model M_1_) significantly outperformed the null model (ΔΧ^2^ = 28.703, *p* < 0.001). BI-RADS score was a robust independent predictor (β = 3.727, SE = 0.973, Wald = 14.67, *p* < 0.001), with higher scores markedly increasing the likelihood of malignancy (Table 2). The model exhibited excellent fit and explanatory power, with a Nagelkerke R^2^ of 0.640 and an AUC of 0.877.

**Figure 5:**
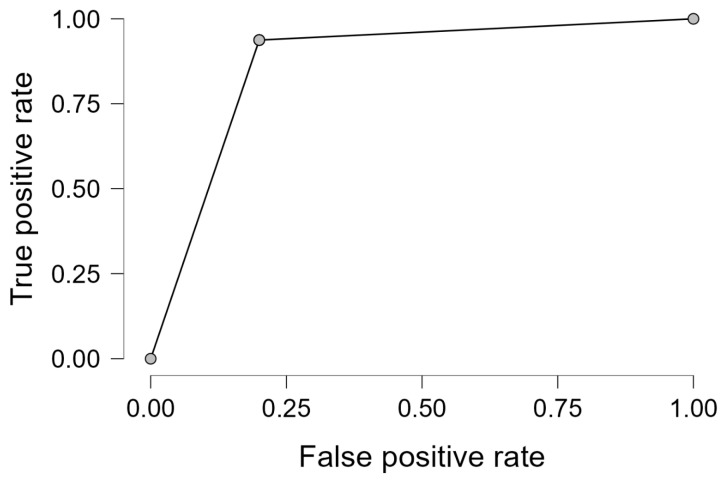
Receiver operating characteristic (ROC) curve for the logistic regression model using BI-RADS score to predict malignancy. The AUC of 0.877 underscores the model’s strong classification performance.

Performance diagnostics revealed an overall classification accuracy of 89.4%, with sensitivity and specificity of 93.8% and 80.0%, respectively. These results indicate that the model is effective not only in detecting malignancy but also in minimizing false positives. The ROC curve in Figure 5 demonstrates the high discriminative capability of the logistic model.

Finally, Spearman’s rank correlation analysis confirmed a statistically significant association between BI-RADS score and binary malignancy outcome (ρ = 0.759, *p* < 0.001), corroborating the previous regression findings and further establishing the clinical relevance of the BI-RADS classification in lesion stratification.

**Figure 6:**
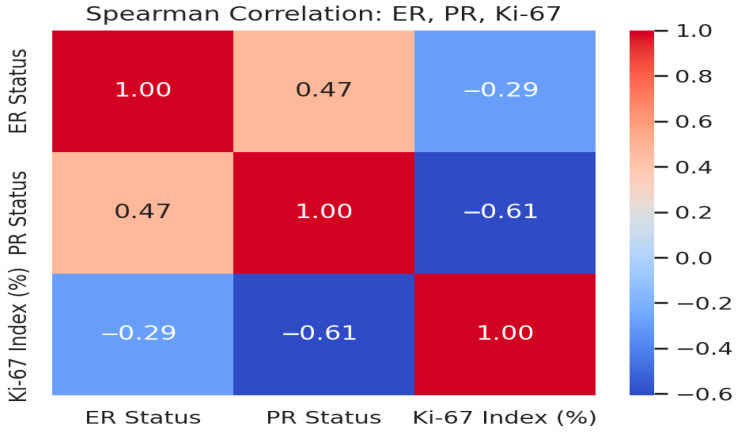
Spearman correlation matrix among ER status, PR status, and Ki-67 proliferation index. A moderate inverse correlation was observed between Ki-67 and hormone receptor expression, indicating increased proliferation in receptor-negative tumors. Analysis is based on a limited subset (n = 14) with available Ki-67 data. Interpretation should be considered exploratory.

In a subset of cases where immunohistochemical data were available, additional analyses were performed to explore the correlation between hormonal receptor status (ER, PR), the Ki-67 proliferation index, and BI-RADS scores. A moderate positive correlation was observed between ER and PR status (Spearman’s ρ = 0.47), while Ki-67 showed an inverse relationship with both ER (ρ = −0.29) and PR (ρ = −0.61), suggesting that high proliferative activity is more common in hormone receptor-negative tumors. These findings are summarized in Figure 6, which presents the correlation matrix among the three variables.

**Figure 7:**
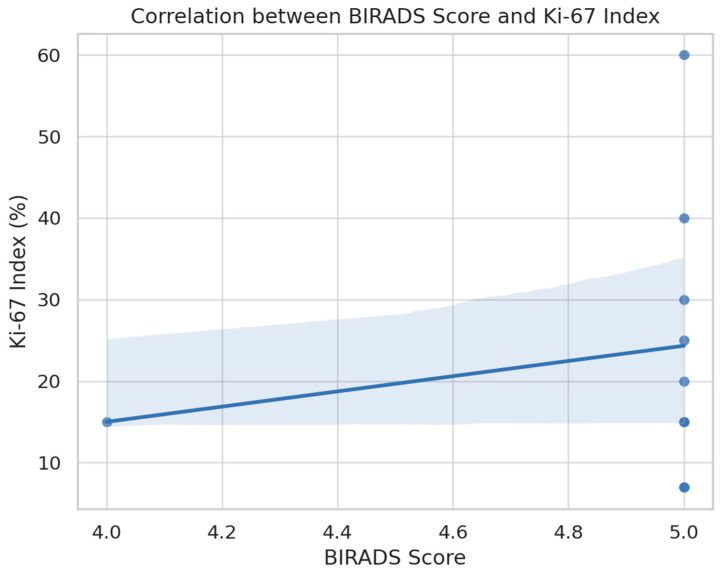
Scatter plot with regression line showing the correlation between BI-RADS score and Ki-67 index. A trend toward higher Ki-67 values in BI-RADS 5 lesions is visible, reflecting a potential link between radiological suspicion and tumor proliferation.

Further, a positive association was noted between BI-RADS scores and the Ki-67 index, as visualized in Figure 7. Although limited by small sample size, the data suggest a trend whereby higher BI-RADS categories are accompanied by increased tumor proliferation. This observation reinforces the biological plausibility of BI-RADS as a surrogate for underlying tumor aggressiveness.

Taken together, the results demonstrate a clear and consistent pattern: radiologic suspicion, as reflected by BI-RADS scores, is strongly associated with both histologically confirmed malignancy and higher tumor grades. Additionally, older age appears to correlate with increased diagnostic severity. The logistic regression model supports the predictive value of BI-RADS scoring and highlights its potential utility in enhancing diagnostic accuracy and clinical decision-making in breast cancer evaluation.

## 4. Discussions

The results of this prospective study confirm the substantial diagnostic value of the BI-RADS classification in stratifying breast lesions and predicting malignancy. The association between BI-RADS scores and histopathological confirmation of malignancy observed in our cohort aligns with findings from multiple studies. Several prior studies have reported similar trends in the predictive value of BI-RADS, particularly for categories 4 and 5. Mohapatra et al. and Aziz et al. demonstrated that BI-RADS 4 subcategories often carry substantial overlap in positive predictive values, underscoring the need for improved stratification within this group [3,4]. Our results corroborate this issue, with some BI-RADS 4 lesions proving malignant, but our data further refine this by correlating BI-RADS with Ki-67 expression, an element absent from earlier studies. Compared to retrospective studies that rely on image-pathology databases, such as those by Mohan et al. and Kuo et al., our prospective design offers enhanced control over timing, data integrity, and consecutive inclusion criteria [5,6]. Importantly, while prior works largely omit immunohistochemical correlations, our study links BI-RADS suspicion directly to proliferation indices and hormone receptor status. This strengthens the potential for imaging to act as a surrogate for biological behavior in resource-limited settings. A key limitation in the previous literature is the exclusion of inconclusive diagnoses and limited reporting on missing data. We address this by transparently reporting and excluding such cases from inferential statistics. Additionally, while prior population-based studies such as Timmers et al. evaluated BI-RADS utility in Dutch screening programs [7], our findings add context from a Romanian tertiary-care setting post-screening era, reflecting real-world diagnostic heterogeneity. This transition from a structured, population-based screening initiative to an opportunistic screening model may have influenced the composition of our cohort. Specifically, patients who self-referred or presented due to symptoms might have had a higher baseline risk, potentially inflating the malignancy rate and the predictive performance of BI-RADS observed in our study. Although this design reflects real-world diagnostic practice, it introduces a potential selection bias that should be considered when interpreting the external validity of our findings. Future studies could stratify patients based on referral source or symptomatology to better delineate these effects. High positive predictive values (PPVs) for BI-RADS 5 lesions have been consistently reported in the literature, underscoring its diagnostic utility [3,4,5]. Our finding that 91% of BI-RADS 5 lesions were malignant confirms the strength of this correlation. A similar impact of BI-RADS 5 classification on clinical decision-making has been demonstrated in population-based studies [6]

Radiologic–pathologic concordance is a cornerstone of breast cancer diagnosis and management. Accurate imaging assessment not only facilitates early detection but also informs treatment decisions and minimizes unnecessary interventions. Correlating imaging findings with histological reports ensures diagnostic precision, as shown by studies examining concordance in BI-RADS 4 lesions and their histological counterparts [4,8,9,10]. When discrepancies occur between imaging and biopsy results, follow-up strategies such as additional imaging, repeat biopsy, or surgical excision are crucial to reduce the risk of underdiagnosis [11,12].

Findings from mammography in our study frequently included calcifications, masses, and architectural distortion. In cases of invasive carcinoma, spiculated margins were a common radiologic finding. Microcalcifications, especially linear or pleomorphic types, were typically associated with ductal carcinoma in situ (DCIS), while developing asymmetry, particularly when associated with architectural distortion or a corresponding mass on ultrasound, was highly suggestive of invasive ductal or lobular carcinoma. These observations reinforce the diagnostic importance of pattern recognition in mammographic interpretation. Ultrasound characteristics further supported lesion stratification. Invasive ductal carcinomas frequently appeared as irregular, spiculated, hypoechoic masses, sometimes accompanied by posterior acoustic shadowing—a feature suggestive of desmoplastic reaction. In contrast, mucinous carcinomas occasionally presented as well-circumscribed or even cystic lesions, mimicking benign features, and underlining the importance of biopsy even in apparently benign cases.

Our analysis also demonstrates a robust logistic regression model, with an AUC of 0.877, sensitivity of 93.8%, and specificity of 80.0%, consistent with the diagnostic accuracies reported by other studies utilizing combined mammography and ultrasound [13,14]. These performance metrics support the integrated imaging approach, which is especially valuable in dense breast tissue where mammography alone may be insufficient [15].

Selective use of breast MRI was instrumental in our diagnostic algorithm, particularly for staging, assessing multifocality or multicentricity, and evaluating dense breast tissue. DCIS frequently manifested as non-mass enhancement on MRI, and invasive lesions were associated with rapid initial enhancement followed by washout kinetics—hallmarks of malignancy in contrast-enhanced breast imaging. A meta-analysis has underscored the discriminative value of MRI BI-RADS descriptors for non-mass enhancement [16]. Such MRI features are aligned with prior data reporting high PPVs for BI-RADS MR classifications in non-mass enhancement [17]. MR imaging patterns have also been associated with specific molecular subtypes, further supporting imaging’s prognostic role [18]

Age emerged as a significant demographic factor, with older patients more frequently diagnosed with malignant lesions. This aligns with large-scale screening data and clinical studies suggesting age as a critical risk factor in breast neoplasia [19,20,21]. BI-RADS scoring, when interpreted alongside patient age and imaging findings, becomes a potent tool for risk stratification. Molecular and immunohistochemical profiling further enhances the understanding of tumor biology. Our correlation between BI-RADS scores and Ki-67 index supports findings that link higher imaging suspicion with aggressive biological behavior [22,23]. Additionally, the inverse relationship between hormone receptor expression and proliferation index reflects established molecular patterns observed in aggressive breast tumors [7,23]. This suggests that imaging features may offer early insights into tumor phenotype even before biopsy confirmation. Our anatomopathological evaluations included invasive ductal carcinoma, invasive lobular carcinoma, DCIS, and mucinous carcinoma. Histological grading followed the Nottingham system (grades 1–3), and the presence of lymphovascular invasion was noted as a prognostic marker of more aggressive disease. Immunohistochemistry was used to assess estrogen receptor (ER), progesterone receptor (PR), HER2 status, and Ki-67 proliferation index, all of which contributed to defining tumor subtype and guiding therapeutic decisions. The higher the Ki-67 index, the more aggressive the tumor behavior, consistent with international findings.

Accurate correlation between imaging and pathology not only confirms diagnosis but also influences surgical planning and guides neoadjuvant therapy decisions. The screening program in which our hospital participated for three years played a major role in early detection, leading to an increase in patient presentations post-program. The visibility of this initiative also enabled the development of a dedicated senology service, where patients presented for symptoms such as skin retraction, pain, nipple discharge, or for asymptomatic screening.

We employed a systematic diagnostic approach that included clinical evaluation, mammography, ultrasound, and tomosynthesis, followed by core needle biopsy using a 14-gauge needle. Regardless of age group, the presence of a palpable breast mass was taken seriously, and the diagnostic triad—clinical exam, imaging, and biopsy—was performed in a stepwise, confirmatory process. Ensuring concordance across these methods is essential for accurate diagnosis and appropriate treatment planning.

From a population health perspective, the transition from a national organized screening program to opportunistic screening offered a unique view into real-world diagnostic workflows. National-level insights, such as those from the Dutch screening program [7], emphasize the importance of consistent imaging standards like BI-RADS in ensuring diagnostic continuity across care settings.

Despite its strengths, BI-RADS is not without limitations. Studies have highlighted the challenges of interobserver variability and subjective interpretation, particularly in borderline categories [24,25,26]. Standardized training and protocol adherence are necessary to minimize these discrepancies. Furthermore, artificial intelligence (AI) and computer-aided detection (CAD) systems are increasingly being explored to support radiologist decision-making and improve reproducibility [27,28,29,30,31]. Recent developments in multimodal deep learning fusion models have shown promise in combining ultrasound and mammography data for optimal BI-RADS-based diagnosis [32]. Although not the focus of this study, such technologies hold promise in enhancing the interpretative consistency of BI-RADS classifications. Our findings resonate with emerging research exploring radiogenomic correlations, where imaging markers align with molecular subtypes and genetic alterations [22,33,34]. Such integrative approaches are paving the way toward personalized imaging diagnostics. Techniques like ultrasound elastography also show promise in refining the evaluation of BI-RADS 3 and 4a lesions [35,36], potentially reducing unnecessary biopsies.

Finally, the cost-effectiveness of breast cancer screening modalities, including digital mammography, has been addressed in several evaluations, highlighting the importance of balancing diagnostic accuracy with resource utilization [37,38,39]. Our data, derived from a Romanian tertiary center, add regional context to this global conversation, contributing to the validation of BI-RADS utility across healthcare systems.

Limitations of our study include a relatively small sample size and limited immunohistochemical data. Longitudinal follow-up for recurrence or survival outcomes was not included and represents an area for future research. Furthermore, external validation in other centers and expansion of the dataset to include additional markers such as HER2 or genomic scores would strengthen the clinical applicability of our findings.

In conclusion, our results reaffirm the clinical robustness of BI-RADS in breast lesion risk stratification, support its correlation with biological aggressiveness, and highlight its continued relevance in multimodal imaging strategies. By bridging imaging features with pathology and molecular biology, this work advocates for integrated, personalized diagnostic pathways in breast cancer care.

## 5. Conclusions

This study highlights the strong correlation between BI-RADS classification and histopathological outcomes in breast lesion assessment. BI-RADS 5 was highly predictive of malignancy, and specific imaging features—such as spiculated margins and pleomorphic calcifications—were consistent with invasive and in situ carcinomas.

Ultrasound and MRI provided valuable diagnostic details, particularly in dense breasts and complex cases. The association between BI-RADS scores and Ki-67 index suggests that imaging may offer insight into tumor biology.

By integrating imaging with histopathology and immunohistochemistry, our diagnostic approach supported accurate risk stratification and informed management decisions. These findings reinforce the value of structured imaging protocols in breast cancer detection and care.

## Figures and Tables

**Table 1:** Cross-tabulation of BI-RADS scores and histological diagnostic categories (n = 47). A strong association was observed between high BI-RADS scores and confirmed malignancies. Histologic categories were grouped for statistical analysis as follows: non-proliferative lesions (e.g., simple adenosis, fibrocystic changes), proliferative lesions (e.g., usual ductal hyperplasia), atypical/malignant potential lesions (e.g., ADH, LCIS), malignant neoplasms (e.g., invasive carcinoma), and inconclusive findings (e.g., insufficient sampling or non-diagnostic material).

BIRADS Score	Atypical Lesions/Lesions with Malignant Potential	Benign Non-Proliferative Lesions	Benign Proliferative Lesions	Inconclusive Diagnoses	Malignant Neoplasms	Total
BIRADS 3	0	1	3	0	0	4
BIRADS 4	0	0	7	1	2	10
BIRADS 5	1	0	1	1	30	33
Total	1	1	11	2	32	47

**Table 2:** Logistic regression results for predicting malignant diagnosis using BI-RADS score as a predictor.

Model	Variable	Estimate	SE	Wald	*p*-Value
M_1_	Intercept	−16.323	4.526	13.01	<0.001
	BI-RADS Score	3.727	0.973	14.67	<0.001

## Data Availability

The datasets generated during the current study are available from the corresponding author on reasonable request. Plans are underway to deposit anonymized data in an institutional repository in alignment with open science practices.

## Abbreviations

The following abbreviations are used in this manuscript:

Abbreviation	Definition
AUC	Area Under the Curve
BI-RADS	Breast Imaging Reporting and Data System
CAD	Computer-Aided Detection
DCIS	Ductal Carcinoma In Situ
ER	Estrogen Receptor
G	Grade (e.g., G1, G2, G3—Nottingham histological grading)
HER2	Human Epidermal Growth Factor Receptor 2
IHC	Immunohistochemistry
KI-67	Proliferation Index Marker
LLM	Large Language Model
MRI	Magnetic Resonance Imaging
PPV	Positive Predictive Value
PR	Progesterone Receptor
ROC	Receiver Operating Characteristic
US	Ultrasound
